# Triage for Potential Percutaneous Coronary Intervention During the Coronavirus Disease 2019 (COVID-19) Pandemic

**DOI:** 10.3389/fmed.2020.567598

**Published:** 2020-10-08

**Authors:** Qi Liu, Sen He, Tian-Yuan Xiong, Yong Peng, Jia-Fu Wei, Chen Li, Ye Zhu, Li Zhang, Mian Wang, Hua Wang, Ming-Xia Zheng, Yun Bao, Ya-Li Wang, Yong He, Mao Chen

**Affiliations:** Department of Cardiology, West China Hospital, Sichuan University, Chengdu, China

**Keywords:** percutaneous coronary intervention, coronavirus disease 2019, infectious, triage, workflow

## Introduction

Since the outbreak of coronavirus disease 2019 (COVID-19), this public health emergency has caused 5,701,337 infections and 357,688 deaths (1). As one of the first few affected countries, China has gradually gained control of the emergency. The West China Hospital of Sichuan University is a regional public health center located in Chengdu, Sichuan province, which mainly provides medical services for southwest China. Our center is also a certified COVID-19 tertiary care hospital and designated hub center within the Sichuan province. The daily volume of our center is ~80,000 visits. For better management of patients with chest pain and those who need percutaneous coronary intervention (PCI), our Department of Cardiology and Chest Pain Center issued a workflow for PCI at the beginning of COVID-19 pandemic. From January 1, 2020, to May 31, 2020, over 4,300 PCIs were performed. However, there were no hospital-acquired COVID-19 cases.

## PCI Workflow and Patient Triage

**Figure 1:**
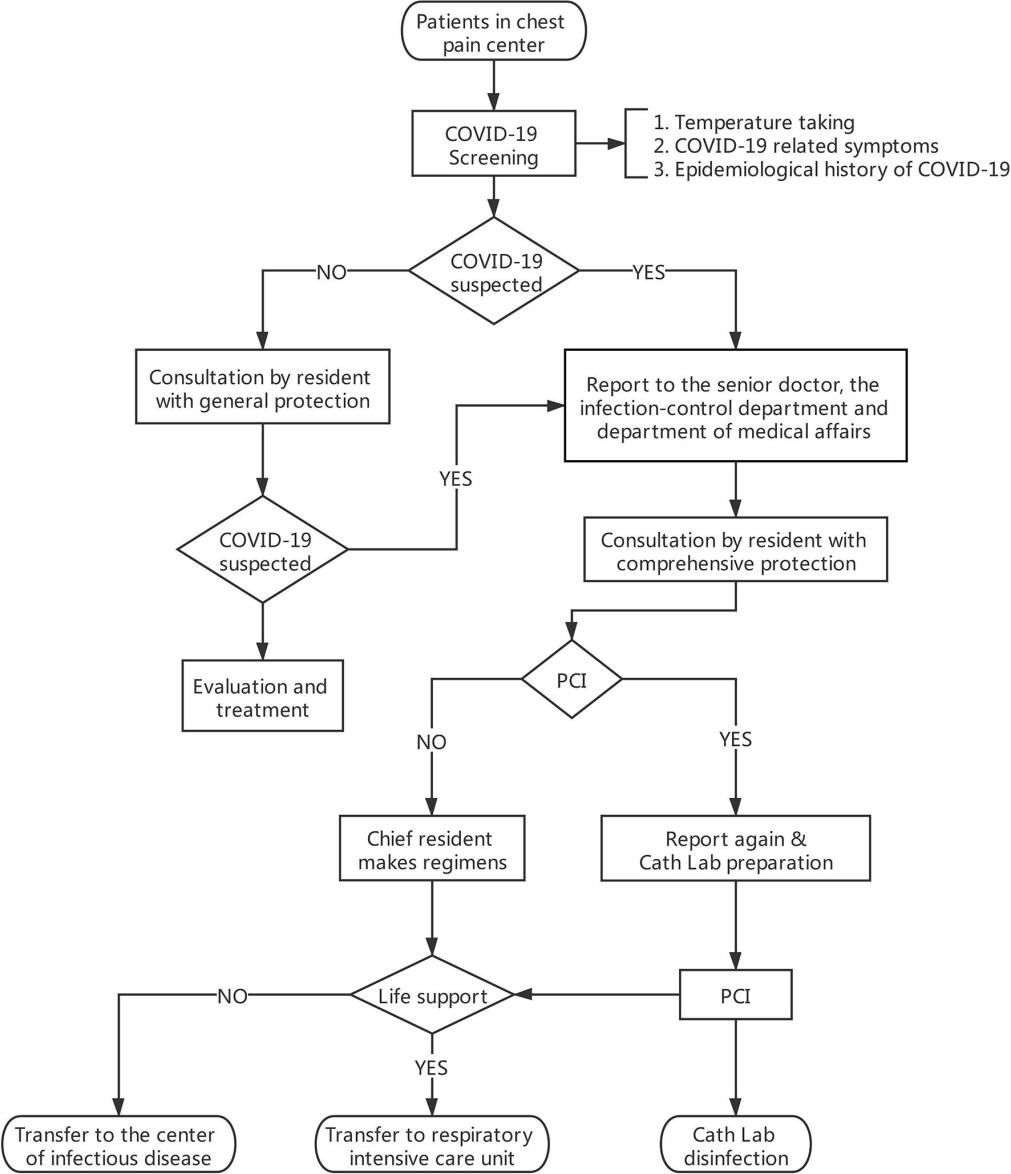
PCI workflow and patient triage process. General protection: medical uniforms, surgical mask, medical cap, and rubber gloves. Comprehensive protection: gown, medical N95 mask, goggles, medical cap, and rubber gloves.

In our medical center, the Chest Pain Center is attached to the Department of Emergency for better management of patients with chest pain, especially those who need emergency medical intervention. Once patients who present with chest pain come to our Chest Pain Center, their vital signs and health status will be evaluated within 10 min. If cardiac emergencies, including acute coronary syndrome, bradyarrhythmia, severe myocarditis, or other situations needing endovascular intervention, are considered, patients will be enrolled in our PCI workflow and patient triage process. The workflow consists of three steps and is presented in Figure 1.

### Step 1. Tele-Communication

The resident, chief resident, and attending-on-shift of the Cardiac Catheterization Laboratory (Cath Lab) are required to tele-communicate with residents of the Department of Emergency and get the exact information of the enrolled patients. The information includes temperature, epidemiological history of COVID-19, and COVID-19-related symptoms (i.e., fever, cough, fatigue, etc.). Based on the information, patients are classified into two categories, non-suspected COVID-19 patients and suspected COVID-19 patients, according to the Novel Coronavirus-Pneumonia diagnostic criteria and treatment regimens defined by the National Health Commission of the People's Republic of China (2).

### Step 2. Consultation

The medical consultation is carried out by the resident of Cath Lab. The consultation route from Cath Lab to Chest Pain Center is set and managed by our center.

For non-suspected COVID-19 patients, consultation doctors are required to wear medical uniforms, surgical mask, medical caps, and rubber gloves. The COVID-19-related information will be checked again, and the temperature will be re-taken. PCI will be carried out in non-suspected COVID-19 patients if endovascular intervention is deemed necessary.

For suspected COVID-19 patients, a reminder will be generated on the consultation system. Consultation doctors ares required to fetch gowns, medical N95 masks, goggles, medical caps, and rubber gloves, and put on the personal protective equipment before the consultation. If PCI is not necessary, a medical regimen and transfer plan to the Center of Infectious Disease or Respiratory Intensive Care Unit (RICU) should be made. If emergent thrombolysis is required, the chief resident should instruct the thrombolysis in the negative-pressure ward of the Chest Pain Center. A further transfer plan to the Center of Infectious Disease or RICU should then be made. When PCI is deemed necessary, consultation doctors should report to the attending-on-shift and the director. Additionally, a report to the Infection-Control Department and Department of Medical Affairs is required so that the hospital can react immediately if any unexpected situation takes place. Then the suspected COVID-19 patient can be sent to the Cath Lab.

### Step 3. Transfer and Procedure

Before the transfer of suspected COVID-19 patients, all medical workers are required to wear a full set of protective equipment including apron, N95 mask, goggles, and rubber gloves,. The protective equipment is available at the Cath Lab. Only after the staff are equipped with protection is the transfer administrated. The transferring route (including the elevator), Cath Lab, and the entrance are previously set. After the procedure, patients who do not need further life support will be transferred to the Center of Infectious Disease, while patients who need further life support are transferred to the RICU. Additionally, the patients' families and companions will be evaluated by the Infection-Control Department and appropriate isolated measures will be taken. Comprehensive disinfection is carried out after the procedure.

## Discussion

Since the outbreak of COVID-19, public health systems worldwide have been confronted with great challenges on medical supply and hospital management. Certain adaptations must be made in the face of COVID-19 (3). During the pandemic, some reforms and suggestions have been made (4). Some centers have also designed crisis management plans (5). An optimized and effective management of PCI workflow will also help control the spread of COVID-19.

Our center is located in Chengdu, where medical resources are not overwhelmed. With the implementation of the proposed workflow, no hospital-acquired COVID-19 has been reported in our Chest Pain Center or Cath Lab. Centers that share similar situations with us may find it useful to implement our workflow. A better management of patients with chest pain will help in this world pandemic.

## Author Contributions

YH and MC had the idea for this article. QL, SH, and T-YX contributed to the writing of the manuscript. YP, J-FW, CL, YZ, LZ, MW, HW, M-XZ, YB, and Y-LW participated in the workflow design and update. All authors contributed to the article and approved the submitted version.

## Conflict of Interest

The authors declare that the research was conducted in the absence of any commercial or financial relationships that could be construed as a potential conflict of interest.

## References

[1] World Health Organization Coronavirus Disease 2019 (COVID-19) Situation Report - 130. Available online at: https://www.who.int/docs/default-source/coronaviruse/situation-reports/20200529-covid-19-sitrep-130.pdf?sfvrsn=bf7e7f0c_4 (accessed May 30, 2020).

[2] National Health Commission of the People's Republic of China Novel Coronavirus-Pneumonia Diagnostic and Treatment Regimens. Available online at: http://www.nhc.gov.cn/yzygj/s7653p/new_list.shtml (accessed May 30, 2020).

[3] King JS Covid-19 and the need for health care reform. N Engl J Med. (2020) 382:e104 10.1056/NEJMp2000821 32302074

[4] Phua J Weng L Ling L Moritoki E Chae ML Jigeeshu VD. Intensive care management of coronavirus disease 2019 (COVID-19): challenges and recommendations. Lancet Respir Med. (2020) 8:506–17. 10.1016/S2213-2600(20)30161-2 32272080 PMC7198848

[5] Stephens EH Dearani JA Guleserian KJ David MO James ST Carl LB. COVID-19: crisis management in congenital heart surgery. Ann Thorac Surg. (2020) 110:701–6. 10.1016/j.athoracsur.2020.04.001 32302660 PMC7194669

